# Supplementary material for: A novel perovskite oxide chemically designed to show multiferroic phase boundary with room-temperature magnetoelectricity
Source: Nat Commun. 2016 Sep 28;7:12772. doi: 10.1038/ncomms12772 (PMC5052705; doi:10.1038/ncomms12772)
Supplement: Supplementary Information — Supplementary Figures 1-6, Supplementary Tables 1-3, Supplementary Notes 1-4 and Supplementary References [file ncomms12772-s1.pdf]

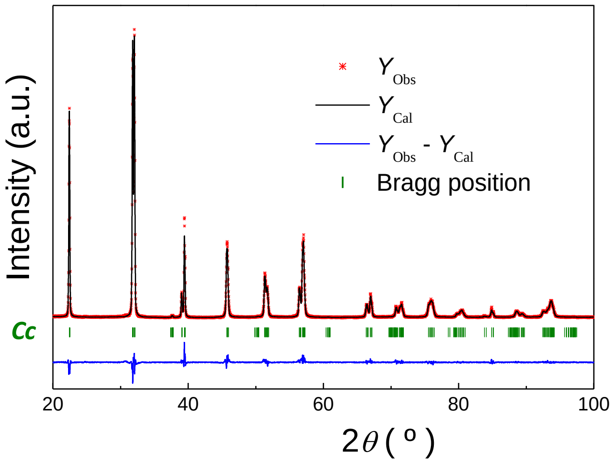

Supplementary Figure 1. Crystal structure for composition $Bi_{0.75}Pb_{0.25}Fe_{0.7}Mn_{0.05}Ti_{0.25}O_3$. High-resolution X-ray diffraction pattern and Rietveld refinement using *Cc* symmetry in a powdered sample at room temperature, for which only one polymorph can be observed. Y_{Obs} and Y_{Cal} refer to the measured and calculated intensities, respectively.

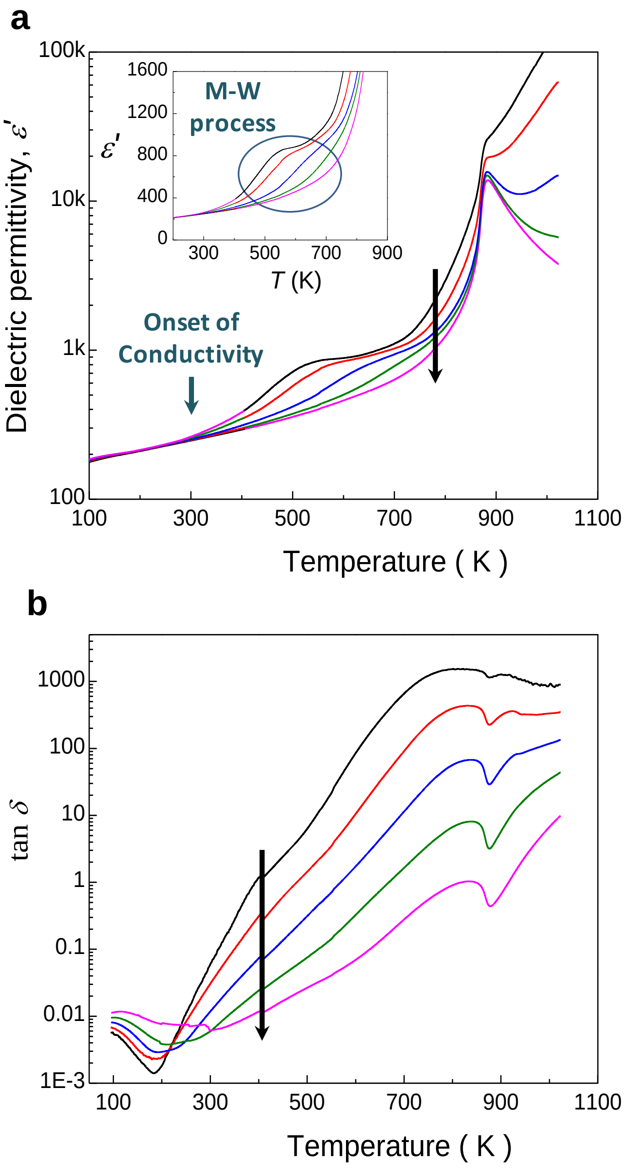

Supplementary Figure 2. Dielectric properties of the $Bi_{0.68}Pb_{0.32}Fe_{0.655}Mn_{0.025}Ti_{0.32}O_3$ ceramic. Temperature dependence of the (**a**) real dielectric permittivity and (**b**) dielectric looses (tan δ) at several frequencies (0.1, 1, 10, 100, 1000 kHz; black arrows indicate increasing frequency). The Maxwell-Wagner type dielectric relaxation is highlighted in the inset (using linear scale in ε'). The onset of electrical conduction takes place above room temperature.

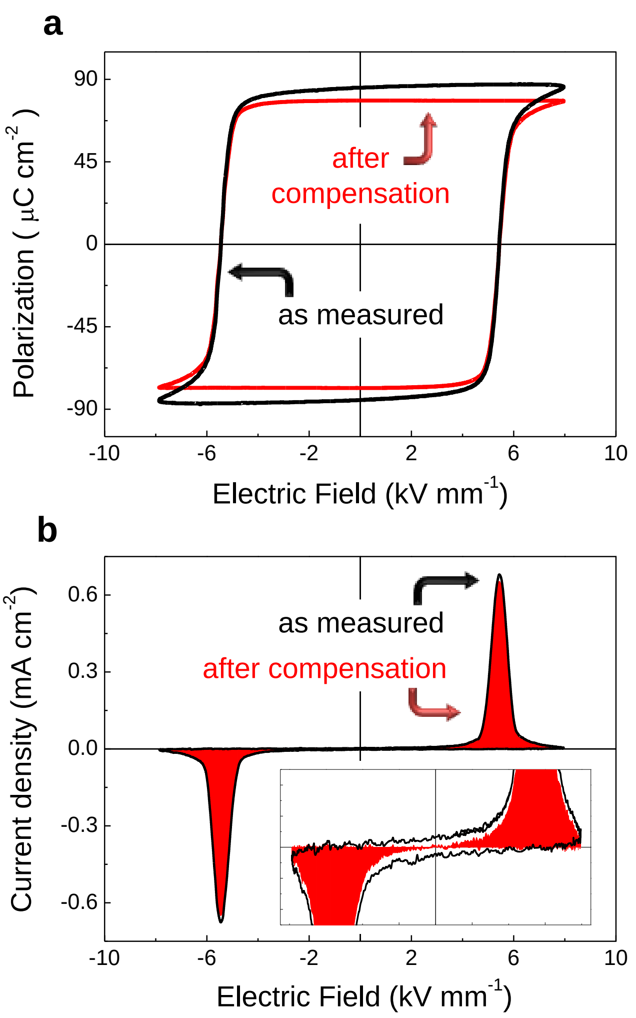

Supplementary Figure 3. Ferroelectric properties of the $Bi_{0.68}Pb_{0.32}Fe_{0.655}Mn_{0.025}Ti_{0.32}O_3$ ceramic. (**a**) **P-E** hysteresis loops and (**b**) current density curves both as measured (black solid line) and after compensation (in red) from linear polarization and conduction contributions (*i.e.*, by subtracting the response of a parallel *RC* component that correctly described the low electric field response). This is a standard procedure that allows isolating the truly ferroelectric switching from artifacts.

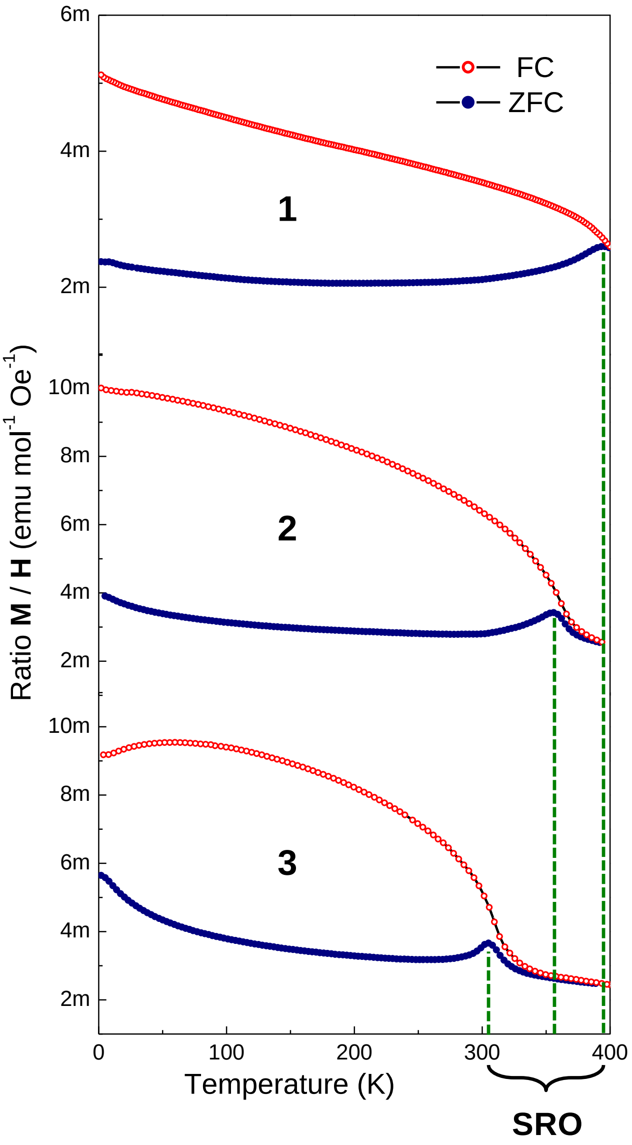

Supplementary Figure 4. Magnetization curves of several compositions. Zero-field-cooled (ZFC) / Field-cooled (FC) magnetization curves under a *dc* magnetic field of 500 Oe of powder samples with composition *x* ($0.4BiMnO_3$-$0.6PbTiO_3$) - (1-*x*) ($0.7BiFeO_3$-$0.3PbTiO_3$) and *x* equal to (**1**) 0, (**2**) 0.0625 and (**3**) 0.15. The material under study $Bi_{0.68}Pb_{0.32}Fe_{0.655}Mn_{0.025}Ti_{0.32}O_3$ corresponds to (**2**) *x* = 0.0625. The temperature at which the spin reorientation (SRO) phenomenon takes place shifts towards lower temperatures with increasing $BiMnO_3$ content, indicating the ability of tuning it along the solid solution.

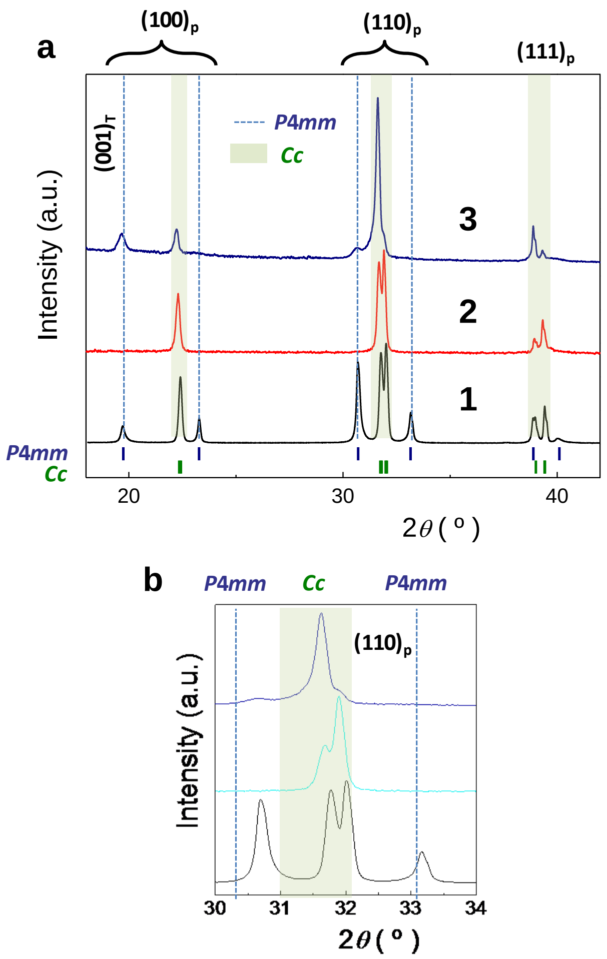

Supplementary Figure 5. Direct characterization of the irreversible field-driven phase change upon poling. (**a**) X-ray diffraction patterns at room temperature of the $Bi_{0.68}Pb_{0.32}Fe_{0.655}Mn_{0.025}Ti_{0.32}O_3$ composition in (**1**) powder sample (for comparison), and those for (**2**) unpoled and (**3**) poled ceramics, along with peak positions for the *Cc* and *P*4*mm* symmetries. (**b**) The region across the $(110)_p$ reflections (referring to pseudocubic indices) is amplified to show the shift of the *Cc* peaks towards lower 2θ angles, indicating an expansion of the monoclinic lattice upon poling the ceramic.

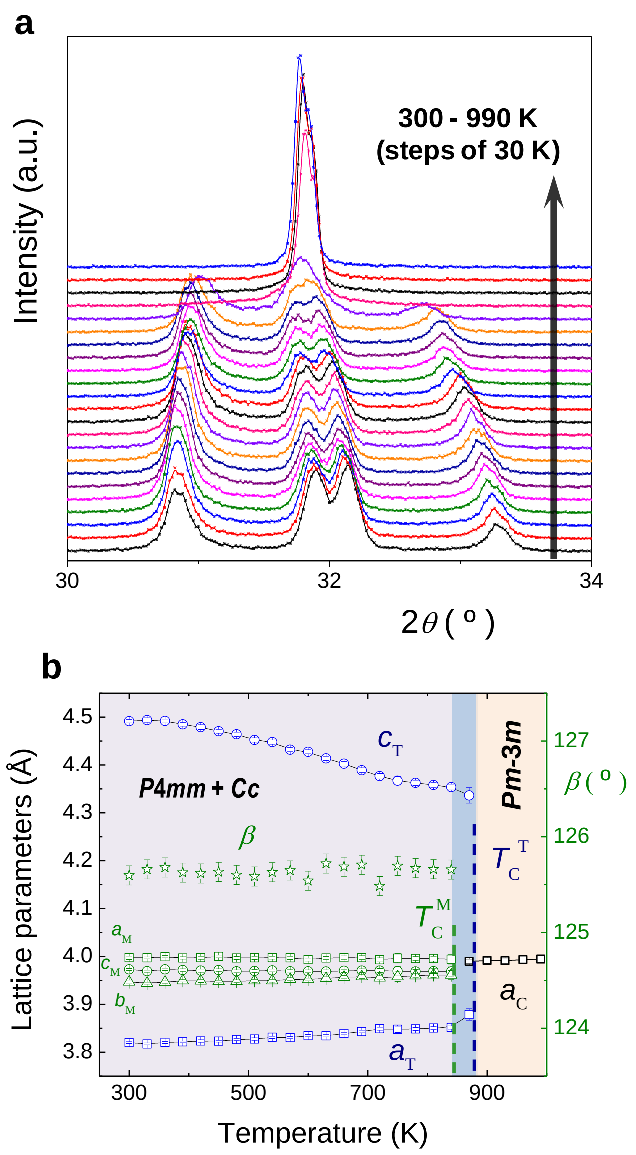

Supplementary Figure 6. Phase evolution with temperature for composition $Bi_{0.68}Pb_{0.32}Fe_{0.655}Mn_{0.025}Ti_{0.32}O_3$. (**a**) Temperature evolution of the X-ray diffraction profiles for powder sample (black arrow indicates increasing temperature from 300 to 990 K in steps of 30 K), indicating phase coexistence of monoclinic *Cc* and tetragonal *P*4*mm* polymorphs. (**b**) Temperature evolution of the lattice parameters for monoclinic (a_M, b_M, c_M, β), tetragonal (a_T, c_T) and cubic (a_C) phases, indicating direct phase transitions of each ferroelectric polymorph to the high-temperature cubic *Pm*-3*m* phase. Curie temperatures for *Cc* (T_C^M) and *P*4*mm* (T_C^T) phases are indicated.

Supplementary Table 1. Refined structural parameters and agreement factors for composition $Bi_{0.68}Pb_{0.32}Fe_{0.655}Mn_{0.025}Ti_{0.32}O_3$. Results of the Rietveld refinements using mixture models with coexistence of rhombohedral *R*3*c* and tetragonal *P*4*mm*, and of monoclinic *Cc* and tetragonal *P*4*mm*. Position coordinates (*x*, *y*, *z*), thermal parameters (B_{eq}), reliability factors (*R*-factors) R_{wp}, R_p, R_{exp} and goodness-of-fit indicator (GOF) are given.

Space group	*R*3*c*			*P*4*mm*			
	Bi/Pb	Fe/Ti	O1	Bi/Pb	Fe/Ti	O1	O2
x	0	0	0.2066	0	0.5	0.5	0.5
y	0	0	0.3388	0	0.5	0	0.5
z	0.3292	0.0894	0.0833	0	0.5703	0.6558	0.1689
B_{eq} (Å^2)	$B_{11} = B_{22}$= 1.14 B_{33} = 0.78 B_{12} = 0.57	$B_{11} = B_{22}$= 0.01 B_{33} = 0.75 B_{12} = 0.01	1.50	$B_{11} = B_{22}$= 2.22 B_{33} = 1.48	$B_{11} = B_{22}$ = 0.90 B_{33} = 2.24	1.50	1.50
Lattice parameters (Å)	*a* = 5.5883; *c* = 13.8438			*a* = 3.8185; *c* = 4.4943			
Statistical parameters	R_{wp} = 10.86; R_p = 7.97; R_{exp} = 3.80; R_{Bragg}(*R*3*c*) = 10.31; R_{Bragg}(*P*4*mm*) = 8.84 **GOF = 2.86**						
Phase fraction	0.54(*R*3*c*) / 0.46(*P*4*mm*)						

S.G.	*Cc*					*P*4*mm*			
	Bi/Pb	Fe/Mn/Ti	O1	O2	O3	Bi/Pb	Fe/Ti	O1	O2
x	0	0.2752	0.0469	0.3201	0.2856	0	0.5	0.5	0.5
y	0.25	0.2539	0.2720	0.4584	-0.0137	0	0.5	0	0.5
z	0	0.7310	0.4623	0.0347	-0.0508	0	0.5530	0.6833	0.1680
B_{eq} (Å^2)	1.39	0.01	1.50	1.50	1.50	2.04	0.76	1.50	1.50
Lattice parameters (Å)	*a* = 9.7914; *b* = 5.5829 *c* = 5.6240; *β* = 125.69					*a* = 3.8188 *c* = 4.4946			
Statistical parameters	R_{wp} = 8.29; R_p = 6.15; R_{exp} = 3.79; R_{Bragg}(*Cc*) = 4.84; R_{Bragg}(*P*4*mm*) = 7.61 **GOF = 2.19**								
Phase fraction	0.56(*Cc*) / 0.44(*P*4*mm*)								

Supplementary Table 2. Refined structural parameters and agreement factors for composition $Bi_{0.75}Pb_{0.25}Fe_{0.7}Mn_{0.05}Ti_{0.25}O_3$. Results of the Rietveld refinements using rhombohedral *R*3*c* and monoclinic *Cc* symmetries. Position coordinates (*x*, *y*, *z*), thermal parameters (B_{eq}), reliability factors (*R*-factors) R_{wp}, R_p, R_{exp} and goodness-of-fit indicator (GOF) are given.

Space group	*R*3*c*		
	Bi/Pb	Fe/Mn/Ti	O1
x	0	0	0.1254
y	0	0	0.3517
z	0.2848	0.0581	0.0833
B_{eq} (Å^2)	0.71	0.01	1.50
Lattice parameters (Å)	*a* = 5.5865; *c* = 13.8429		
Statistical parameters	R_{wp} = 12.12; R_p = 8.97; R_{exp} = 9.18; R_{Bragg} = 6.06 **GOF = 1.32**		

Space group	*Cc*				
	Bi/Pb	Fe/Mn/Ti	O1	O2	O3
x	0	0.2751	0.0442	0.3275	0.2830
y	0.25	0.2431	0.2917	0.4536	-0.0275
z	0	0.7310	0.4602	0.0337	-0.0491
B_{eq} (Å^2)	0.93	0.01	1.50	1.50	1.50
Lattice parameters (Å)	*a* = 9.7634; *b* = 5.5893; *c* = 5.6347; *β* = 125.8584º				
Statistical parameters	R_{wp} = 11.19; R_p = 8.41; R_{exp} = 9.17; R_{Bragg} = 4.25 **GOF = 1.22**				

Supplementary Table 3. Crystal spontaneous polarization for $Bi_{0.68}Pb_{0.32}Fe_{0.655}Mn_{0.025}Ti_{0.32}O_3$.

Calculation of the spontaneous polarization from the ion displacements provided by the crystal structure refinements of the $Bi_{0.68}Pb_{0.32}Fe_{0.655}Mn_{0.025}Ti_{0.32}O_3$ powder sample for both monoclinic *Cc* and tetragonal *P*4*mm* structures. Average effective charges (z'') for the A- and B-cations, lattice volume (V), ferroelectric shifts ($\delta_{A\text{-}O}$ and $\delta_{B\text{-}O}$) of the A-site and B-site cations, respectively, as compared to the cubic phase and determined from structural refinement, and the crystal spontaneous polarization (P_S).

**Space group**	z''	V (Å^3)	$\delta_{A\text{-}O}$ (Å)	$\delta_{B\text{-}O}$ (Å)	P_S (μC cm^{-2})
Monoclinic ***Cc***	A-site = 4.8<br>B-site = 4.5	249.69	0.563	0.249	98
Tetragonal ***P4mm***		65.55	0.755	0.316	123

Supplementary Note 1. Rietveld refinement of the $Bi_{0.68}Pb_{0.32}Fe_{0.655}Mn_{0.025}Ti_{0.32}O_3$ structure.

The space group *P*4*mm* (No. 99) was considered as initial structural model for the tetragonal phase. In this model, the Bi/Pb occupy the Wickoff position 1a site at (0, 0, δ_z), the Fe/Mn/Ti occupy the 1*b* site at (½, ½, ½+δ_{zb}), and the oxygen occupy two different Wyckoff positions: O1 is placed at 1*b* site at (½, ½, δ_{zO1}) and O2 in 2*c* site at (½, 0, ½+δ_{zO2}). The Bi/Pb was settled at the origin of the unit cell[1,2].

For the rhombohedral phase, a hexagonal distortion of the ideal perovskite with space group *R*3*c* (No. 161) and $a = \sqrt{2}a_p$ and $c = 2\sqrt{3}a_p$ (a_p -parameter of the primitive perovskite unit) was considered[3]. The Wyckoff positions are as follow: cations located at A and B positions correspond to a 6*a* Wyckoff position, whereas the Oxygen is located at the 18*b* Wyckoff position. The coordinates considering this hexagonal unit cell would be Bi/Pb = (0, 0, ¼+*s*), Fe/Mn/Ti = (0, 0, *t*) and O =(1/6-2*e*-2*d*, 1/3-4*d*, 1/12). The *s* and *t* parameters are involved in the displacement of A-site and B-site cations in the perovskite. Oxygen octahedral tilt angle ω is related with the parameter *e* thought the expression tan $\omega = 4\sqrt{3}\ e$, the octahedral distortion is shown in the *d* value, whereas the octahedral strain is related with the formula (1+ ζ). The initial values of $BiFeO_3$ were chose for the refinements[4].

Finally, monoclinic *Cc* space group (No. 9) with $a = \sqrt{6}\ a_P$, $b = \sqrt{2}\ b_P$, $c = \sqrt{2}\ c_P$ was considered. In this case, the 4*a* Wyckoff position generates five atoms and their coordinates are as follows: Bi/Pb at (0, ¼, 0), Fe/Mn/Ti at (¼+δ_x, ¼+δ_y, ¾+δ_z), O1 at (0+δ_{x1}, ¼+δ_{y1}, ½+δ_{z1}), O2 at (½+δ_{x2}, ½+δ_{y2}, 0+δ_{z2}) and O3 (½+δ_{x3}, 0+δ_{y3}, 0+δ_{z3}). The initial parameters were taken from previously reported data[5].

Supplementary Note 2. Irreversible field-driven phase change upon poling in the $Bi_{0.68}Pb_{0.32}Fe_{0.655}Mn_{0.025}Ti_{0.32}O_3$ ceramic sample at room temperature.

X-ray diffraction (XRD) patterns at room temperature of the unpoled and poled ceramic of the composition under study is given in Supplementary Fig. 5. Experiments were performed on the same ceramic sample before and after poling the material at high electric fields (9 kV mm^{-1}). The irreversible field-driven phase change upon poling is unambiguously demonstrated. Before the poling step, the ceramic is basically monoclinic, while its pattern is strongly modified upon poling. Note firstly, the appearance of the tetragonal $(001)_T$ and $(101)_T$ reflections at about 19.7º and 30.7º in 2θ, respectively, demonstrating the phase-change phenomenon to take place. Note also the presence of an underlying domain texture within the tetragonal phase, for the $(100)_T$ and $(110)_T$ reflections (*i.e.*, peaks splitting corresponding to *P*4*mm*) are not observed. This indicates that the new tetragonal phase nucleates with polarization aligned along a crystallographic direction as close as possible to the poling electric field (perpendicular to the sample´s major surfaces), as one would expect.

A similar domain texturing takes place in the remaining monoclinic *Cc* phase. The splitting characteristics of the $(110)_p$ and $(111)_p$ doublets is drastically modified, indicating an increased fraction of domains with polarization aligned along the allowed crystallographic direction nearest to the poling field. Taking into account that non-180º domain walls of both ultrahigh tetragonal and rhombohedral/ monoclinic $BiFeO_3$-based systems are basically immobile, this indicates the phase-change to involve the monoclinic fraction that is initially unfavorably oriented with the poling field. Mechanism would be nucleation and movement of antiphase boundaries. Note that some stress-driven relaxation is expected after removing the electric field, which otherwise is necessary for reversible field-induced phase change to take place. Note also that additional phase changes during subsequent polarization reversal are not expected, for it is thought to be controlled by 180º switching within the new mixed-phase configuration.

Supplementary Note 3. Born effective charges and calculation of crystal polarization.

Effective charges (z'') used for the calculation of the crystal spontaneous polarization were taken from the literature. They have been calculated by Hewat[6] and experimentally verified in many ferroelectric compounds (*e.g.*, $PbTiO_3$)[7], and more recently in $BiFeO_3$ by several groups to predict their huge crystal polarization[8-10]. Effective charges used in our calculations were: Pb^{2+} = 3.6; Ti^{4+} = 5.7; O^{2-} = -3.1 (calculated by charge neutrality in ABO_3: z'' A + z'' B + 3z'' O = 0); while Bi^{3+} = 5.4 (assuming an isotropic value $xx = yy = zz$) and Fe^{3+} = 3.9 (this one calculated by charge neutrality assuming the same isotropic values for O^{2-} = -3.1 as in $PbTiO_3$).

All the atoms in $BiFeO_3$ and $PbTiO_3$ present large z'' values that exceed the nominal ionization charges by a factor near 2 at most. Mn content was too low, so it was neglected and assumed as Fe. So, as the shifts of all the A-site cations are the same (and *idem* for the B-site cations), this leads to averages z'' for A- and B-cation effective charges of: z'' = 0.68 x 5.4 + 0.32 x 3.6 = 4.8 (A-site cations) and z'' = 0.68 x 3.9 + 0.32 x 5.7 = 4.5 (B-site cations).

For a *k*-ion with an effective z''_k charge and a δ_k displacement from cubic position, the local polarization is given by z''_k x δ_k and the total polarization is:

$$P_S = \frac{e}{V} N \sum_{\kappa} z''_{\kappa} \delta_{\kappa}$$

where *e* is the electron charge, *V* the lattice volume (obtained also from structural analysis) and *N* the number of molecule/dipole per unit: *P*4*mm* = 1 and *Cc* = 4. Data to calculate the crystal polarization in both *Cc* and *P*4*mm* structures are summarized in Supplementary Table 3.

Supplementary Note 4. Direction of the polarization and of the tilting of oxygen octahedra.

In the *P*4*mm* phase polarization is pointing along the pseudo-cubic *c*-axis, while in the *Cc* phase the structure is of the $a_+^- a_+^- c_+^-$ type, according to Stokes tables[11]. This means that polarization has two positive and equal components along its ⟨100⟩ and ⟨010⟩ axes, and one positive different component along the ⟨001⟩ axis, while oxygen rotates along $a^- a^- c^-$ (two equal angle-components along ⟨100⟩ and ⟨010⟩ axes and one different angle-component along ⟨001⟩ axis, all of three in anti-phase). The *Cc* phase has its axis along ⟨110⟩, ⟨110⟩ and ⟨112⟩ of the pseudo-cubic phase[12].

In our case, the polarization carried by the Fe/Ti (B-site displacements) is mainly along the monoclinic *a*-axis, with a weak (8%) component along *b*-axis, and a zero component along *c*-axis, whereas the polarization carried by Bi/Pb (A-site displacements) is also along monoclinic *a*-axis, with a zero component along *b*-axis and a less weak (18%) component along *c*-axis. So the resulting polarization is mainly along monoclinic *a*-axis, that is, along ⟨110⟩ of the pseudo-cubic phase at room temperature (of course, polarization direction is temperature dependent).

Regarding the antiferrodistortive rotations in the *Cc* phase, in a octahedra rigid-model of the structure, the axis about which the oxygen octahedra tilt is along a [uuv] direction due to group theory relationships, *i.e.*, with two equal rotations along the ⟨100⟩ and ⟨010⟩ pseudo-cubic axes, and another component with a different angle along the ⟨001⟩ pseudo-cubic axis. However, distortions of oxygen octahedra are also allowed by symmetry, and in this compound they are so high and prevalent that oxygen atom positions are not resulting from a simple rotation which should have clearly indicated an actual rotation axis.

Supplementary References

1 Shirane, G., Pepinsky, R. & Frazer, B. C. X-ray and neutron diffraction study of ferroelectric $PbTiO_3$. *Acta Crystallogr.* **9,** 131-140 (1956).

2 Kothai, V., Senyshyn, A. & Ranjan, R. Competing structural phase transition scenarios in the giant tetragonality ferroelectric $BiFeO_3$-$PbTiO_3$: Isostructural vs multiphase transition. *J. Appl. Phys.* **113**, 084102 (2013).

3 Megaw, H. D. & Darlington, C. N. W. Geometrical and structural relations in the rhombohedral perovskites. *Acta Crystallogr.* **A31**, 161-173 (1975).

4 Fischer, P., Polomska, M., Sosnowska, I. & Szymanski, M. Temperature dependence of the crystal and magnetic structures of $BiFeO_3$. *J. Phys. C: Solid State Phys.* **13**, 1931-1940 (1980).

5 Bhattacharjee, S., Senyshyn, A., Fuess, H. & Pandey, D. Morin-type spin-reorientation transition below the Néel transition in the monoclinic compositions of $(1-x)BiFeO_3$-$xPbTiO_3$ (x = 0.25 and 0.27): A combined *dc* magnetization and x-ray and neutron powder diffraction study. *Phys. Rev. B* **87**, 054417 (2013).

6 Hewat, A. W. Soft modes and the structure, spontaneous polarization and Curie constants of perovskite ferroelectrics: tetragonal potassium niobate. *J. Phys. C: Solid State Phys.* **6**, 1074-1084 (1973).

7 Malibert, C. *et al*. Order and disorder in the relaxor ferroelectric perovskite $PbSc_{1/2}Nb_{1/2}O_3$ (PSN): comparison with simple perovskites $BaTiO_3$ and $PbTiO_3$. *J. Phys.: Condens. Matter* **9**, 7485-7500 (1997).

8 Ravindran, R., Vidya, R., Kjekshus, A. & Fjellvåg, H. Theoretical investigation of magnetoelectric behavior in $BiFeO_3$. *Phys. Rev. B* **74**, 224412 (2006).

9 Goffinet, M., Hermet, P., Bilc, D. I. & Ghosez, Ph. Hybrid functional study of prototypical multiferroic bismuth ferrite. *Phys. Rev. B* **79**, 014403 (2009).

10 Dixit, H. *et al*. Understanding strain-Induced phase transformations in $BiFeO_3$ thin films. *Adv. Sci.* **2**, 1500041 (2015).

11 Stokes, H. T., Kisi, E. H., Hatch, D. M. & Howard, C. J. Group-theoretical analysis of octahedral tilting in ferroelectric perovskites. *Acta Cryst.* **B58**, 934-938 (2002).

12 Hatch, D. M. *et al*. Antiferrodistortive phase transition in $Pb(Ti_{0.48}Zr_{0.52})O_3$: Space group of the lowest temperature monoclinic phase. *Phys. Rev. B* **65**, 212101 (2002).
